# Barriers to maternal retention in HIV care in Ghana: key differences during pregnancy and the postpartum period

**DOI:** 10.1186/s12884-020-03067-8

**Published:** 2020-07-17

**Authors:** Kwame S. Sakyi, Margaret Y. Lartey, Caitlin E. Kennedy, Julie A. Dension, Luke C. Mullany, Prince G. Owusu, Emma Sacks, Emily A. Hurley, Pamela J. Surkan

**Affiliations:** 1 Department of Public and Environmental Wellness, Oakland University, School of Health Sciences, 3101 Human Health Building, 433 Meadow Brook Rd, Rochester, MI 48309-4452 USA; 2 Center for Learning and Childhood Development-Ghana, AF 3190 Adenta Flats, Accra, Ghana; 3 Department of International Health, Johns Hopkins Bloomberg School of Public Health, 615 N. Wolfe Street, Baltimore, MD 21205 USA; 4 Department of Medicine & Therapeutics, University of Ghana School of Medicine & Dentistry, CHS, P.O. Box GP 4236, Accra, Ghana; 5 Health Services and Outcomes Research, Children’s Mercy, 2401 Gillham Road, Kansas City, MO 64108 USA

**Keywords:** Retention in HIV care, Ghana, Qualitative, Postpartum, Pregnancy

## Abstract

**Background:**

Maternal retention in HIV care is lower for women in the postpartum period than during pregnancy, but the reasons are poorly understood. We examined key differences in barriers to retention in HIV care during and after pregnancy.

**Methods:**

We conducted semi-structured, in-depth interviews with 30 postpartum women living with HIV. Participants were recruited from two tertiary facilities implementing Option B+ for prevention of mother-to-child HIV transmission in Accra, Ghana. We collected data from mothers who had disengaged from HIV care and those who were still engaged in care. The interviews were analyzed using principles adapted from grounded theory.

**Results:**

Participants’ experiences and narratives showed that retention in HIV care was more challenging during the postpartum period than during pregnancy. Poor maternal physical health (from birth complications and cesarean section), socio-cultural factors (norms about newborn health and pregnancy), and economic difficulties linked to childbirth (such as unemployment, under-employment, and debt) made the costs of retention in HIV care more economically and socially expensive in the postpartum period than during pregnancy. Some participants also shared that transportation costs and resulting dependence on a partner to pay increased during the postpartum period because of a strong shift in maternal preference for private modes of transportation due to HIV stigma and the desire to protect the newborn. These factors played a larger role in the postpartum period than during pregnancy and created a significant barrier to retention. A conceptual model of how these factors interrelate, the synergy between them, and how they affect retention in the postpartum period is presented.

**Conclusions:**

In Ghana, lower retention in HIV care in the postpartum period compared to in pregnancy may be primarily driven by social, economic, and newborn health factors. Multifaceted economic-based and stigma-reduction interventions are needed to increase retention in maternal HIV care after childbirth.

## Background

The United Nations Sustainable Development Goals 3.3 and 3.7 call for an end to the HIV/AIDS epidemic and universal access to sexual and reproductive health care services by 2030 [1]. Meeting these targets requires retention in HIV care and increased access to antiretroviral therapy (ART) and postpartum health services. In the past two decades, access to ART has dramatically risen in sub-Saharan Africa, particularly among pregnant women [2]. Some countries like Ghana have reduced several key barriers to ART access, including providing ART at no cost and implementing Option B+, where pregnant women are put on lifelong ART, regardless of their CD4 cell counts [3].

Despite these advancements, recent studies demonstrate that after ART initiation, women living with HIV have difficulty remaining in HIV care during the first year after childbirth (referred to henceforth as the postpartum year) [4, 5]. Poor retention in HIV care (missing scheduled visits to collect ART or lost-to-follow-up) in the postpartum year is widespread in both high-and low-income countries. A meta-analysis of retention rates among pregnant and postpartum women in Option B+ programs in low-and middle-income countries showed that retention declined from 90 to 75% between 3 months after HIV diagnosis (typically during pregnancy) and 9–12 months postpartum [5]. In Ghana, only 66% of postpartum women are retained in HIV care [6], compared to 75% among pregnant women [7] and 86% among the general population of adults living with HIV [8]. In South Africa, one study found that about 50% of pregnant women living with HIV dropped out of care in the postpartum year [9].

Poor retention in HIV care is closely associated with interruptions in ART and consequential higher viral load, which increases the risk of mortality, HIV disease progression, HIV transmission to a partner, and mother-to-child HIV transmission (MTCT) [10–12]. At 20%, Ghana has the fourth highest rate of MTCT of HIV among the 23 high burden countries identified by UNAIDS and the second highest in West Africa [13]. The average rate in high burden countries is about 11.8% [13]. One reason for these high rates of MTCT is low retention in the postpartum year [14]. Over half of the cases of HIV transmission to children in high-burden countries occurs during breastfeeding [14]. Thus, improving HIV retention in the postpartum year is critical to global efforts to reduce MTCT of HIV, especially while a mother is actively breastfeeding.

It remains unclear why dropping out of HIV care is more common in the postpartum year than during pregnancy. Two recent systematic reviews on disengagement from HIV care demonstrate that several of the significant barriers to retention—HIV stigma, fear of disclosure, and social support—in the postpartum year are also prevalent during pregnancy [4, 5]. Yet, retention loss is more common in the postpartum year than during pregnancy.

Some have suggested that motivation to remain in care decreases after delivery, as mothers may be less concerned about their children acquiring HIV after delivery than during pregnancy [15, 16]. Another barrier may be that mothers believe that their own HIV care is less important after their babies are born uninfected [17]. One limitation of these studies has been a focus on identifying distinct, individual-level factors. Prior studies may have also overly relied on the perspectives of mothers still in care, instead of those who have disengaged [4, 9]. The results of these studies also highlight the similarity of the barriers between pregnancy and the postpartum year. Given these findings and limitations, what is unique to postpartum women has been difficult to characterize.

In this paper, our primary goal is to contribute to theories about why retention loss is more prominent in the postpartum year than during pregnancy. We used a qualitative approach to explore the experiences of Ghanaian women living with HIV. We describe critical barriers to retention in HIV care by examining how maternal physical health, socio-economic factors, and cultural forces linked to pregnancy and childbirth interact to negatively impact retention.

## Methods

### Setting

This study was conducted in the Greater Accra Region of Ghana. Ghana is a lower-middle-income country with 23.4% of the population living below the national poverty level [18]. HIV prevalence is 2.0% among adults overall and 2.8% among pregnant women [19]. At 3.8%, the prevalence of HIV among women in the Greater Accra Region is the second highest across Ghana’s ten regions [19].

Study participants were recruited from two tertiary hospitals in Accra: Korle Bu Teaching Hospital and Ridge Regional Hospital. Korle Bu is the largest HIV treatment center in the country with about 25,000 patients per year. At both facilities, pregnant women are routinely tested for HIV during antenatal care, and if confirmed HIV-positive, referred to an adult HIV treatment center, called the *Fevers Unit* at Korle Bu and *ART Treatment Center* at Ridge. Adult and pediatric HIV services are provided in separate buildings at both hospitals. Antenatal care and postnatal care are also physically separated from the adult and pediatric HIV services.

**Fig. 1:**
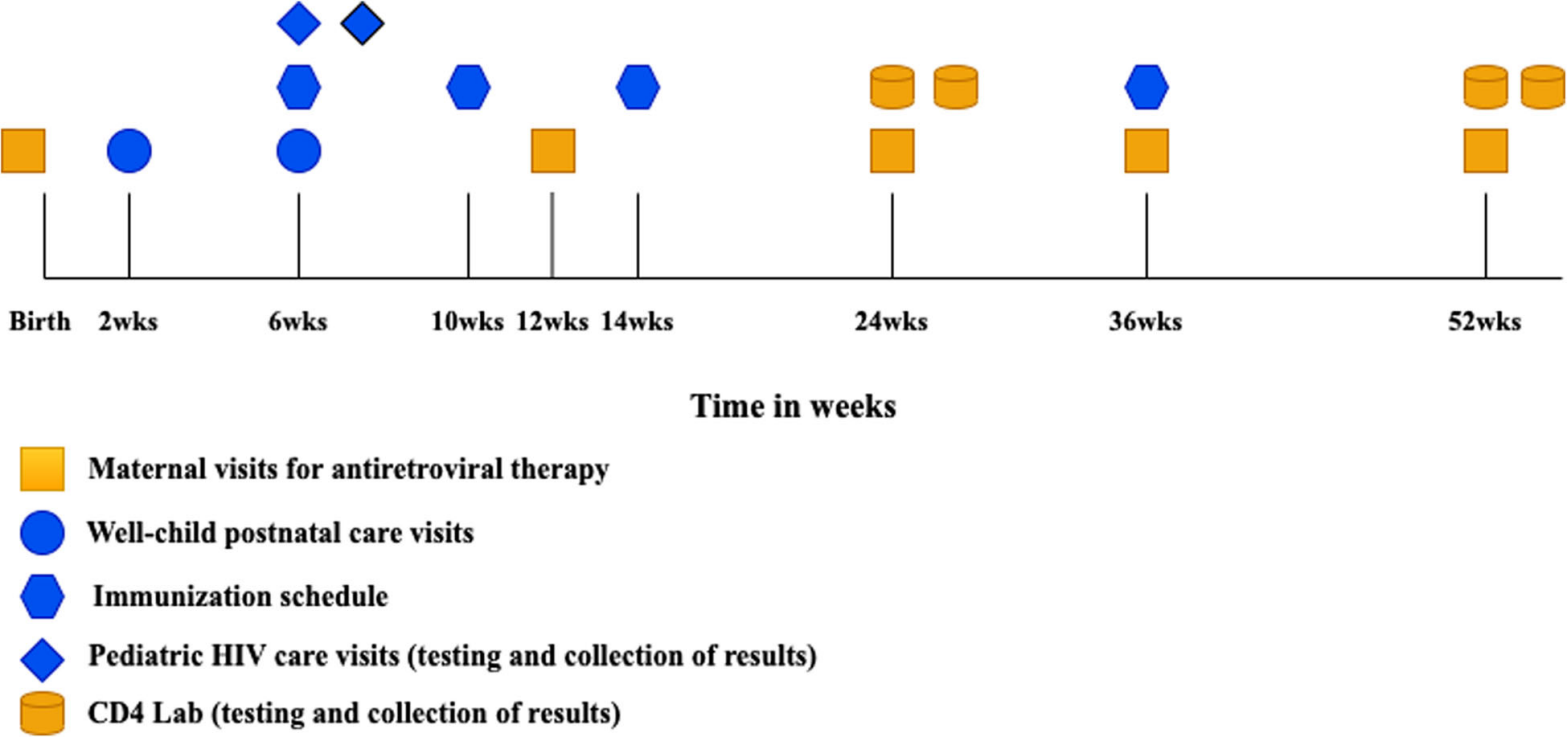
Recommended schedule for HIV, maternal, and child healthcare visits in Ghana in the postpartum year

In Ghana, mothers living with HIV make several separate visits for HIV, maternal, neonatal, and child health services in the first year after giving birth. At both hospitals, free ART is available for patients, which is typically dispensed in three-month intervals. Consequently, to have uninterrupted treatment in the postpartum year, mothers must attend at least four visits at an HIV treatment center throughout the year. These appointments exclude visits for laboratory tests, such as CD4 cell count, which is done every 6 months at the two hospitals and requires two visits per test for sample collection and receiving results. For their children’s health, mothers attend two routine postnatal care visits within the first 6 weeks, in addition to two visits after 6 weeks, three visits for immunizations, one separate visit at 6 weeks for an early infant HIV diagnostic test, and another visit to collect the test results (See Fig. 1) [20, 21].

### Study participants and recruitment

Participants included 30 urban, adult postpartum women (18 years or older) living with HIV who had given birth at one of the two urban hospitals in the 12 months preceding data collection. This article was one of the components of a qualitative project that explored the influence of birthweight, as a marker of infant health, on postpartum retention in HIV care. Thus, mothers in the study were purposefully sampled based on their infants’ hospital-recorded weight at birth (low birthweight (< 2500 g) or normal birth weight (2500 g or greater)). They were also sampled based on time since childbirth (before and after the first 6 months) to examine how the barriers they faced changed over time.

All participants were recruited through health care workers (nurses, midwives, and HIV-related counselors) from hospital records, adult and pediatric HIV treatment clinics, antenatal and postnatal clinics, and neonatal intensive care units. First, participants were recruited through pharmacy medical records. The pharmacies of the two hospitals had the contact information of all mothers living with HIV who gave birth at their hospitals, including their birthweight and date of birth. With permission from the hospital administration, a hospital health worker telephoned the mothers who gave birth and described the study to them. The contact information of those who were interested was forwarded to a female member of the study team, who was a previously trained ART counselor with significant experience working with people living with HIV. Mothers with the most recent deliveries (during first 6 months preceding data collection) were interviewed first. Second, after the initial interviews, more tailored, in-person recruitment of participants was conducted through health workers (using theoretical sampling [22]—determined through initial discussion of themes emerging from the data). In each unit, health workers were briefed about the study’s goal, procedures, and eligibility criteria. Interested patients who met the eligibility criteria (including those who had disengaged from care) were referred to a study team member, who provided more details about the study and enrolled the potential participant. Recruitment stopped when data saturation was reached (i.e. no new reasons for disengagement in care during pregnancy and in the postpartum year were emerging from additional interviews).

### Data collection

Data were collected from February to April 2016. We used qualitative methods informed by grounded theory [22], with the aim of generating theory from the data. The initial interview guide was developed based on our research questions, which explored: (1) experience with childbirth and also living with HIV; (2) experience with retention and ART adherence during labor and immediate postpartum at the hospital; (3) interactions with relatives and friends after childbirth, as they related to their baby’s size and HIV status; and (4) experience with retention in HIV care during pregnancy and the postpartum year. Participants were also asked to share their thoughts on why retention in HIV appeared challenging in the postpartum year compared to during pregnancy.

The final interview guide was developed iteratively. The guide was first used with four participants and then modified. The data collection team role-played with the interview guide and listened to each other’s interviews. This exercise was used to refine the content, structure, and wording of the interview guide and improve the research assistants’ interview techniques. The interview guide has been published elsewhere [23].

The interviews occurred at the recruiting hospitals or in participants’ homes. Two research assistants (male and female) and the first author conducted the interviews in Twi or Ga. The research assistants received a week-long training on the research protocol, interview techniques and ethics. Interviews on average lasted 45 min (range 20 to 95 min). Two women were called for a follow-up interview to further clarify issues discussed in the first interview. All but four interviews were audio-recorded. Handwritten notes were taken in the case that a participant declined to be recorded. Audiotapes were transcribed verbatim and then translated to English. Participants also completed a structured questionnaire regarding socio-demographic characteristics and HIV history.

### Data analysis

Qualitative data analysis occurred concurrently with data collection. After the first seven interviews, the research team met to discuss emerging themes. A working theory was formulated about the major factors serving as barriers to retention. New interviews were then used to confirm, reject, and refine various aspects of the working theory during regular debriefing sessions, as well as to tailor sampling [22]. The interview guide was updated after these initial discussions to allow for a more in-depth examination of key themes.

Following data collection, more formal coding of the transcripts was conducted. Two of the authors, KSS and PGO, developed a codebook based on inductive coding of the first five interviews and concepts that emerged from the debriefing sessions. The first author (KSS) then systematically applied to the rest of codes to the interviews. Codes were modified as needed to clarify boundaries and to ensure the consistency of the coding. Segments of transcripts related to reasons for keeping clinical appointments, prolonged disengagement from care, missed visits, and recommendations for improving HIV care were extracted, and more focused coding was done to identify underlying barriers and strategies. Using the constant comparative method [24], quotes for each theme were compared to others to delineate how they were connected within and across interviews, and between pregnancy and the postpartum year.

Informed by Ware et al. [25], we took an interpretive analytic approach to identify underlying processes beyond listing the key barriers. Analytic memos were written to elucidate themes. The analysis of transcripts was managed using Atlas.ti 7 [26].

### Ethics

All participants provided written informed consent, and each participant received an equivalent of $5 USD in local currency as compensation for time and travel. Additionally, each participant received a package of diapers. The study was approved by three ethics review boards: (1) the Johns Hopkins Bloomberg School of Public Health Institutional Review Board (IRB No. 6651), (2) the Ethical and Protocol Review Boards of the Ghana Health Services (ID: Ms-Et/M.2-P4.1/2015–2016) and (3) the University of Ghana Medical School (ID: GHS-ERC 16/09/15).

## Results

A total of 33 participants were recruited for the study. Three of them declined to participate because they did not have time. On average participants were 35 years old (range 24 to 44 years), had two children (range 1-5), were five months postpartum, and had been on HIV treatment for 3.8 years (Table 1). All but one lived with a partner. About a third of participants had more than a primary school education level, and three-quarters worked as small business traders or dressmakers. Out of the thirty participants, six had disengaged from HIV care in the postpartum year; these participants had been out of care from two to eight months (starting during pregnancy). Eight additional participants had missed at least one HIV care visit in the postpartum year.

**Table 1:** Background characteristics of study participants and their infants, *N* = 30

Characteristics	Total N (%)
**Categorical Variables**	
**Education level**	
No formal education	1 (4)
Primary school	8 (29)
> Primary School	19 (67)
**Marital status**	
Married	24 (80)
Cohabitating	5 (17)
Single/widowed	1 (3)
**Ethnicity**	
Akan	12 (40)
Ewe	7 (23)
Ga	3 (10)
Other	8 (27)
**Occupation**	
Trader	14 (46)
Seamstress	5 (17)
Other	11 (37)
**Time of HIV diagnosis**	
Before index pregnancy	21 (70)
During index pregnancy	8 (27)
During postpartum, index pregnancy	1 (3)
**Continuous Variables**	**Mean (range**
Maternal age (years)	34.80 (24–44)
Number of children	2.09 (1–4)
Number of years living with HIV	4.14 (0.4–16)
Mean birth weight (kg)	2.49 (1–3.5)
Infant age (months)	4.80 (0.17–12)

### Overview of themes

**Fig. 2:**
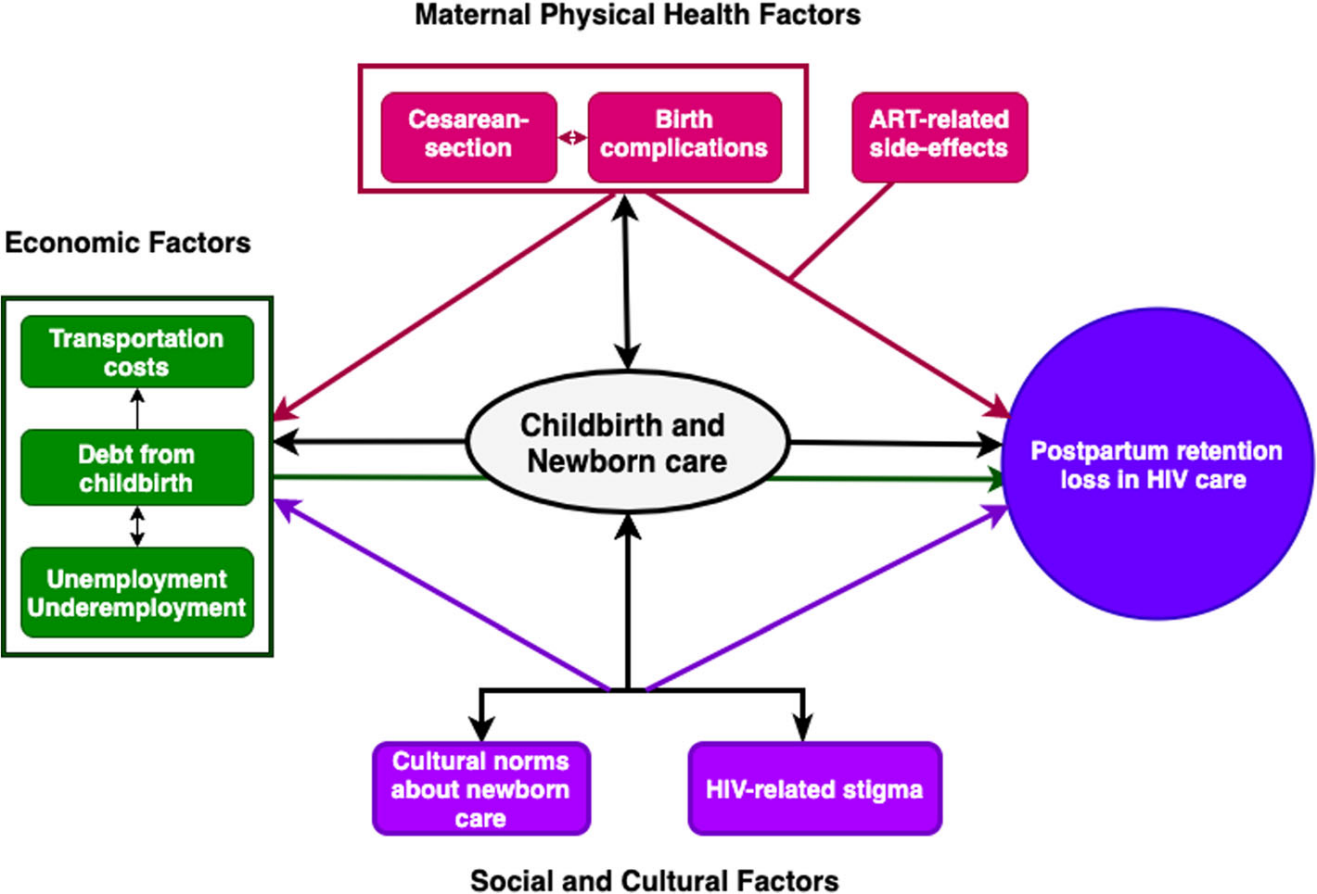
Synergy between physical health, economic, and socio-cultural barriers to postpartum women’s retention in HIV care

Participants’ accounts show that retention in the postpartum year was more difficult than during pregnancy because of strong interactions between maternal physical health, socio-cultural forces, and economic inadequacy linked to childbirth and caring for a newborn. These factors, as the data showed, created conditions where the economic and social costs of staying in HIV care were higher in the postpartum year than during pregnancy. In Fig. 2, we illustrate the synergy between these barriers and how they contribute to retention loss in the postpartum year. Those who had completely disengaged from care indicated experiencing multiple barriers at the same time.

**Fig. 3:**
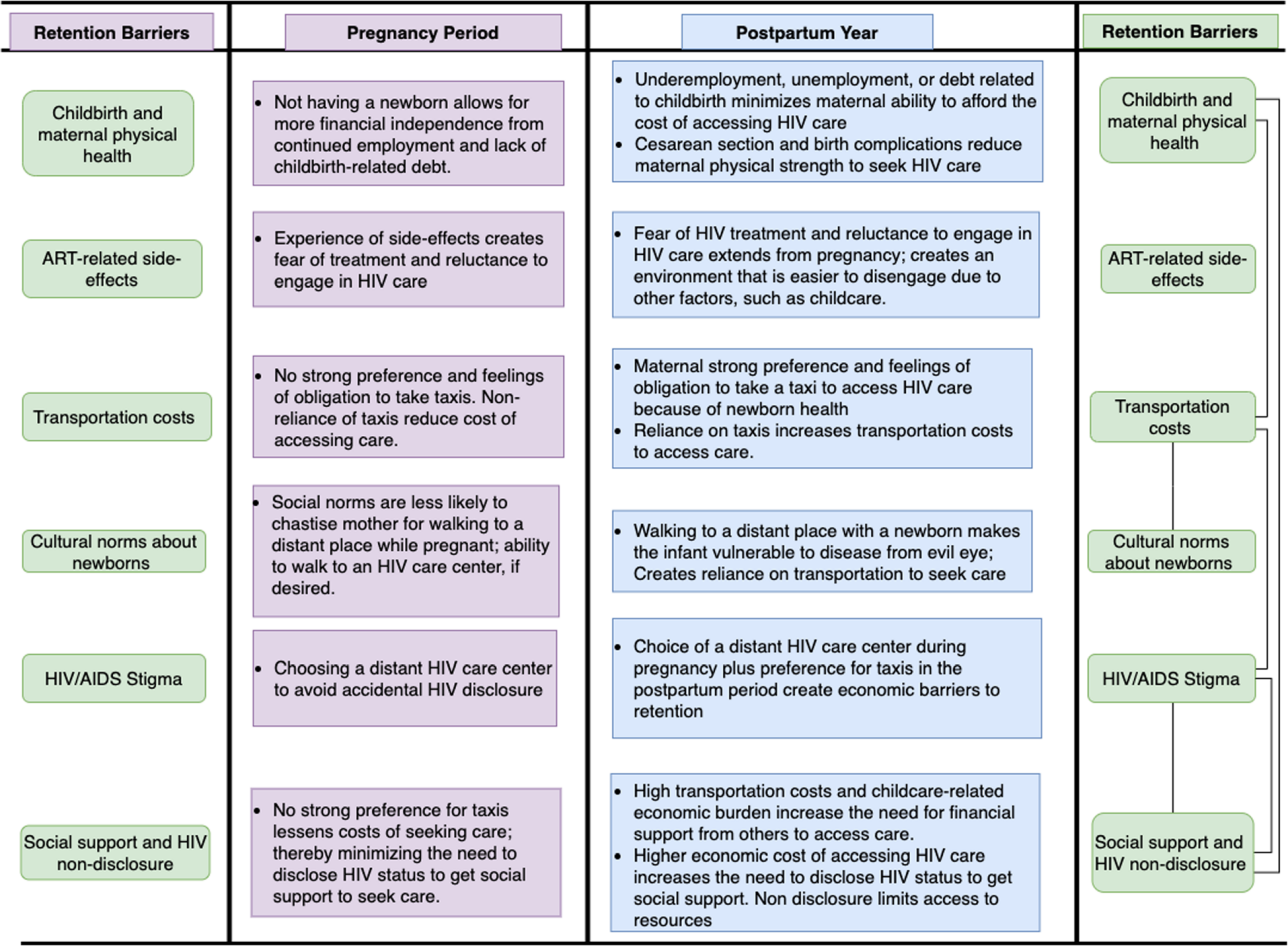
A comparison of retention barriers in HIV care during pregnancy and the postpartum year

Figure 3 compares retention barriers during pregnancy and the postpartum year.

### Maternal physical health

#### Cesarean section and birth complications

Participants explained how physical health problems after childbirth, particularly due to cesarean section and birth complications, made retention in the postpartum year more challenging than in pregnancy. The interviews showed that poor maternal physical health as a barrier to retention was unique to, and more prominent in the postpartum year than during pregnancy. A few of the mothers commented that in the immediate months after childbirth, mothers who had cesarean sections were just not physically strong enough to attend their ART visits, even when their motivation to stay in care was high. For example, one participant who had been out of HIV care for 6 months explained that she suffered from ART side-effects. However, she also cited her physical health after childbirth as contributing to her disengagement.

I could not also go for my [HIV] treatment because, after I delivered, I spent one week at the hospital because I had a high BP (blood pressure). They also saw that I was bleeding through my vagina, so they placed a cotton there … They, however, forgot to remove the cotton, so I became bloated. I was bloated for like four days … [A] nurse, in charge of taking me and my newborn to the Special Clinic [a clinic for pediatric HIV diagnosis and care] saw that my feet were swollen, and I could not walk. She asked whether I had an operation (cesarean section) done and I said no, but I told her that they have not removed the cotton inside me … She rushed me to the hospital ward for them to remove it. It was after that, that I was able to walk well again [Five months postpartum, seven months out of HIV care]*.*

Another participant, who was still engaged in care explained:

Most mothers will tell you that the first six months are very tough. Many women take the time to work on themselves since some of them undergo an operation [i.e., c-section]. Others too had a vaginal tear, so they may need some time to take care of themselves. It takes six months or more for a mother to return to normal life after childbirth [i.e. to be healthy and to visit the HIV treatment centers for care]. Some too are single mothers who have no support at home [Twelve months postpartum, no missed visit]*.*

#### ART-related side effects and reluctance to resume care

ART-related side effects and HIV denial were the most distinctive barriers, comparing mothers who disengaged from HIV care during pregnancy and those who disengaged in the postpartum year. In particular, women who were in denial would describe ART-related side effects as sufficient reason to disengage from care during pregnancy. Once the baby was born, however, denial gave way to determination to keep the infant from becoming HIV infected. For example, one participant, who had denied her HIV infection reported:

I told the nurses that I was having problems [i.e. ART-related side effects]. They told me that is how it is at the beginning. It will go away. I still did not take it after that and [stopped attending my visits]. Then after pregnancy, when I gave birth, the nurse told me to take it [the ART] because of the baby. That is why I am here [to collect my medications]. I would be taking it. I know by the power of God, my baby will not get it [HIV]. I will not stop taking the drugs until the child gets tested, and he is negative. [Two months postpartum; six months out of HIV care]

This relationship between side effects and HIV denial followed a consistent pattern in the data: side effects generally provided an impetus for pregnant and postpartum mothers to disengage when they encountered side effects combined with other barriers, like challenges with childcare, or mistreatment from health workers. For example, one participant narrated that she was diagnosed with HIV during pregnancy and reported starting ART without receiving counseling about the potential side effects: *“They did not take their time to explain the side effects of the drugs to me … When I tested positive, they just put me on the drugs.”* She reported diligently taking ART until one morning, she woke up with a “*swollen mouth”, “sores all over [her] face”,’* and *“swollen red eyes.*” As a result, she disengaged from care for about 7 months, five of which were during the postpartum year. During this period, she was also seeking care from a pastor because she also thought that an “evil eye” was causing her side-effects. When she did re-engage in HIV care at the advice of a health worker, she described how mistreatment from healthcare providers led her to stop seeking HIV care and resuming ART again.

All that while [living with the pastor], I was not taking my medicine [ART]. It was when I gave birth to her that I went to the hospital. They told me that since the child was breastfeeding, I should take the medicine and give the child medicine also [pediatric HIV prophylaxis] for six weeks. Just around that time, we were also moving to Kumasi to live with my mother, so their instruction was that if the medicine got finished, I should go to any hospital [in Kumasi] for a refill. When I went to the hospital in Kumasi however, they did not it give to me. They mistreated me, so I stopped following-up after that. [Six months postpartum; eight months out of HIV care].

A few of the participants reported being mistreated by health workers (for example shouting, yelling, being impatient, and intentionally delaying service to patients). However, only one participant attributed her disengagement from care to the mistreatment, in addition to the side-effects.

All those who had dropped out of care because of ART-related side effects were women who had been newly diagnosed with HIV during pregnancy. A few of the mothers felt that these side effects were related to their pregnancies and made them question the efficacy of the treatment. The fear of re-experiencing the side effects created a reluctance to re-engage in care during the postpartum year, even when their stated motivations to prevent MTCT of HIV was high.

During pregnancy I suffered when I took the drugs due to the side-effects. Thus, anytime I thought of going back for the drugs [after the birth] the thoughts of the side-effects came to my mind and they troubled me. I became afraid. [Five months postpartum, seven months out of HIV care]*.*

### Economic barriers to retention loss

#### Transportation costs

Transportation cost was a common barrier to retention in both pregnancy and the postpartum year. However, it was more frequently cited as a barrier in the postpartum year than during pregnancy. In fact, it was the predominant reason for disengagement in the postpartum year. Participants described that in the postpartum year, particularly during the first 6 months, maternal motivation to ensure newborn well-being obligated them to take a taxi to clinical appointments rather than the cheaper, local bus (called *trotro*^1^*)* that most women took during pregnancy*.* They feared that the close contact with people on the crowded local buses would put the newborn at risk of illness. One participant explained: “*There may be airborne diseases in the trotro since people might be coughing … I did not want her to get infected by any disease.’* Another mother concurred*: “Now that I have delivered, I have to come by taxi, but before, I used to come here [the Fevers Unit] by trotro.”* Thus, the financial cost of accessing HIV care was higher in postpartum than during pregnancy, which hindered the ability of many women to obtain care. For one mother who had been out of care for two months, for example, the cost of transportation to take her baby from where she lived to Korle Bu in a taxi would have cost her about 140 GHC (or $35). This amount was more than five times the cost of taking a *trotro* (24 GHC or $6) and 70% of her monthly salary.

#### Underemployment and unemployment and debt from childbirth

For a few women, the high cost of transportation to stay in care in the postpartum year occurred in the context of childbirth-related debt and inadequate employment. For some of the mothers, particularly those who were self-employed, one reason for higher economic insecurity during the postpartum year, compared to pregnancy, was that they temporarily stopped working to take care of their newborns. For example, one mother was a security officer during pregnancy and earned about 200 GHC a month (about $50 at the time of data collection). She did not, however, have an income at the time of data collection because she took an unpaid maternity leave after birth*.* The lack of adequate income and financial dependence, as explained by participants, led to missed visits because, even when they had high motivation, needed to wait for others to financially support them.

We had financial challenges, so my sister had to go and borrow money to pay [the medical bills] … The man does not take care of me, and I have this child too … I really find it difficult to go for my drugs, but I try so hard to go. If I have to go for refills, I have to take a taxi, and if I do not have the money, I cannot go. [Six months postpartum, unemployed, one HIV care missed visit]

Another woman explained:

At times, we are unable to come to our visits because of poor finances. It is difficult because you might not be working. For instance, I have my own job … I just take my money, pay for transportation, and go to the hospital, even if he [my husband] does not give me money. [Two months postpartum; six months out of HIV care]

Moreover, many of the mothers explained that despite having health insurance, which covered basic childbirth costs, they were required by the hospitals to cover additional costs related to cesarean-section, birth complications, or their child’s health. The debt made it harder for households to access ART in the immediate (first 6 months) postpartum year. The mother who had taken unpaid maternity leave described how her debt impacted her household’s finances. “*I was discharged on the 28th of December, but I did not have money [to pay the hospital bills], so I was still there [at the hospital.]* She continued, *“The man too [has] his job [but it] is not lucrative … In addition, he complains that he has to find money to settle the debt we incurred from borrowing money from others to pay for the hospital bills [for the new baby].”*

### Socio-cultural factors

#### Cultural norms about newborn care, HIV-related stigma

Cultural norms around newborn care (with particular concern for newborn illness) and HIV-related stigma also contributed to more challenges to retention in the postpartum year than during pregnancy. Both pregnancy and the postpartum year constitute a vulnerable time for newborns to be infected with *Asram*, a locally understood illness that is caused by an evil eye [27]. A newborn can be infected in utero or after birth, but the mechanism for this infection is understood to differ between pregnancy and the postpartum year. *Asram* could be caused by a woman wearing shorter skirts, exposing the pregnant stomach, and eating street food during pregnancy [28]. In contrast, in the postpartum year, however, it is exposing the child to many people in public spaces that could result in this illness [29]. As a result, a few of the participants who might have walked to access care during pregnancy also felt obliged to take taxis during the postpartum year, both out of fear of exposing the newborn to illness and fear of being chastised for walking long distances with their baby:

Anytime I take them [the twins] out, people complain that why am I travelling with them to a faraway place. Now that my finances are also down, I have to be picking a taxi with them everywhere I go, and that is also another cost. Since I delivered, I do not have much money on me, so if I consider all the costs involved, then I do not go at all. [Five months postpartum; seven months out of HIV care]

HIV-related stigma also impacted retention loss through its effect on transportation costs and access to financial resources. Some participants said that they intentionally sought HIV care farther away from home during pregnancy to avoid being recognized by their neighbors at the HIV treatment centers. Thus, transportation became even more costly because of the distance and pressure to avoid potential stigma. One postpartum mother explained her decision regarding where to seek care:

They asked me in which suburb of Accra do I live? I told them Kasoa. They suggested two options to me: I could routinely collect my drugs at Korle Bu or Winneba. I told them Korle Bu because if I choose Winneba, people may recognize me because I am a hairdresser [Ten months postpartum; five and half months out of HIV care].

Moreover, a few of the participating women described that they could not ask their partner and relatives for financial support to seek care because they did not want to disclose their HIV status. One mother explained:

One week after I delivered, I took a taxi there [to the HIV treatment center] and she [a nurse] asked me whether there was anyone at home to take care of the baby for me. Because I didn't want anyone to know where I was going, I took him [the baby] along. At the time, my in-laws and my mother were around, and I told them that my husband had asked me to come to his shop … The woman [nurse] was the one who paid for a taxi to take us back home [Three weeks postpartum, one missed visit in HIV care]*.*

### Prioritization of children’s health

Notably, all mothers who disengaged from their own care continued to attend child postnatal visits. Attendance for child visits occurred even when women missed their own visits. Partially driving this commitment to children’s postnatal visits was a concern that all mothers expressed about passing HIV to their children. This was evident in both the interviews and in that all but one mother attended all pediatric HIV testing visits.

Similarly, prevention of MTCT was the dominant reason mothers remained in HIV care during pregnancy. After the child’s birth, almost all women described being strongly motivated to find out if they had prevented transmission. One mother commented *“Why would you want to bring this devilish disease on your child*?” Two mothers who had disengaged from care reported:

I thought it [HIV] would have a negative effect on the child. I was scared she might have the disease [HIV], so … we came, and had his blood tested, and it came out negative. I was glad [Six months postpartum; eight months out of HIV care*.*

Another participating woman explained:

They come [for the child's HIV test] because they want to make sure that the child is well [has not been infected]. If the child is infected, they cannot go to work or do our job. They have to come because of the child. [Two months postpartum; six months out of HIV care]*.*

Women may also have prioritized children’s health due to careful allocation of limited resources; many women attended their children’s postnatal care at community clinics nearby, which meant lower transportation costs compared to going to the hospital for their own care. Furthermore, because the adult HIV and pediatric HIV care facilities were close in proximity to each other, it was easier and less expensive for mothers to receive postnatal and pediatric HIV care in one trip (than to attend either of those visits in addition to their own HIV care visits).

### Participants’ recommendations on how to improve retention

Participants' made health-system related recommendations to improve women’s retention in HIV care. These suggestions centered on allowing community clinics to run CD4 testing (instead of tertiary hospitals—to avoid long queues), and extending intervals between medication refills in the postpartum year (e.g. from three to six months) to reduce visit burden. A quote from one woman illustrates a problem with refills shared by many participants.

At the Police Hospital where I receive my treatment, what they do is that … when you come for your six weeks [well-child postnatal care] visit, they give you more pills to last you for a long time. Only a serious illness might bring you back again … No mother will tell you the first six months is easy to [continue in HIV care] … [so it will help us stay in care] if the number of pills they give us is maybe for six months or more … Some women even travel to their hometown to give birth. Let us say you give that person only a three-months supply of ART and the person too will be spending six months there. When her three month's supply is finished, she will have no choice but to stay another three months without treatment. [One and half months postpartum, no missed visit in HIV Care]

Another woman suggested that it would be helpful if community clinics also did CD4 count testing.

I think the small polyclinics [community clinics] should be equipped to run CD4 tests. Since they do not perform this test, you have to go to Korle Bu. When you get there also, there are so many people there and you have to join a long queue, unless you go there at dawn … I am not breastfeeding him so I can leave him behind and go, but some women cannot do that because these newborns they usually have a weak immune system, they could acquire an infection at the hospital. You also have to make multiple visits to get the test and the results—the back and forth—is really a worry to us. They should allow the polyclinics to do the tests and that will reduce the revisits and the long queues at Korle Bu. [One month postpartum, no missed visit in HIV care]

## Discussion

We found that, in the Ghanaian context, having a newborn created an environment where maternal physical health, socio-economic and cultural norms, and HIV-stigma interacted synergistically to make engagement in HIV care more difficult in the postpartum year than during pregnancy. We found that there were strong dynamic interactions between concern for the newborn’s health, HIV stigma, and transportation costs in the postpartum year. The shift in maternal transportation preferences from public to private options (with its associated costs) after the child’s birth was shaped by socio-cultural forces, and created a situation in which the economic and social burden of staying in HIV care in the postpartum year was more significant than during pregnancy. These high costs were compounded because they also co-occurred in a context where maternal physical health, debt, and underemployment linked to childbirth made households more vulnerable.

This interpretation shifts the focus from individual-level factors [15–17] as reasons for poor retention in the postpartum year and points to interconnected social and economic conditions as a principal explanation. Prior studies have suggested that the disparity in retention loss between pregnancy and the postpartum year is due to changes in maternal motivation to stay in HIV care after delivery [16, 30, 31], less frequent contact with the healthcare system in the postpartum period [4], and maternal beliefs that one’s own HIV care is not as important as the care of the newborn [9]. In the urban part of Ghana, however, we found that even when maternal motivation to stay in HIV care was high and women believed that their health was important, retention loss in the postpartum year persisted. Moreover, we found that women living with HIV in Accra were required to make several (at least 10) visits within the postpartum year for postnatal, child health, pediatric HIV, and adult HIV care-related services. Thus, the hypothesis that higher postpartum retention loss is due to less frequent visits in the postpartum year compared to pregnancy [15] may be less applicable in this setting. All, but one of the participants who had disengaged from HIV care for their own health were still engaged with the health system for routine postnatal care and pediatric HIV care services. Our results suggest that difficult economic conditions, complicated by HIV stigma and newborn care, and fear of re-experiencing medication side effects from medications, may contribute to these disparities in retention rates.

The barriers described in our study are consistent with findings from a recent systematic review of contextual factors affecting ART initiation, adherence, and retention for HIV-infected pregnant and postpartum women [4], which found that across contexts, actual or anticipated stigma, dependence on a partner, and transportation problems were major barriers to retention in the postpartum year [4]. Our study extends these findings by unpacking how socio-economic barriers within the context of caring for a newborn creates further barriers for women during the postpartum year. It is this increased synergy centered around childbirth and newborn care, that makes women more vulnerable to retention loss in the postpartum year than during pregnancy. In addition, our results draw attention to the critical factors that are unique to the postpartum year: the impact of maternal physical health after childbirth (birth complications and cesarean section) and its associated economic costs (under-employment, debt) – but that have been under explored.

Based on these findings, future studies on retention for HIV care in low-income settings among postpartum women should examine interactions between factors influencing care and how they are compounded to produce disparities in retention rates, rather than focusing on individual or even structural barriers alone. Relying on these two approaches, Wawrzyniak et al. examined factors related to retention among adults living with HIV in the United States [32]. By using viral load as a biomedical indicator for poor retention in HIV care, they found that the odds of having a detectable viral load were three times higher among participants who experienced three or more individual level barriers compared to those who did not report any barriers. Similarly, in our study, mothers who disengaged from care experienced multiple barriers that compounded the challenges of staying in care. Future studies might quantitatively explore how the synergistic effects of HIV-related stigma, social support, and transportation problems might explain the disparities in retention rates comparing pregnancy to the postpartum year.

We also found that the experience of ART-related side-effects was a primary reason for discontinuation from HIV care, particularly during pregnancy [33, 34]. Our findings add to the existing literature by providing a more nuanced understanding of how ART-related side-effects impact retention specifically in the postpartum year. In Malawi, Kim et al. [33] found that some mothers disengaged from HIV care because the experience of side effects made them question the efficacy of ART. We further found that the experience of ART-related side effects led some mothers to disengage from care when they encountered other barriers, such as mistreatment from health workers. In the era of Option B+, these findings imply that providing early support for pregnant women who experience ART-related side effects and other barriers is critical, as the effects may be exacerbated in the postpartum year.

Our findings should be considered in the context of our study’s strengths and limitations. Our sample included only urban women who delivered at a health facility and were receiving HIV care at tertiary hospitals, potentially limiting the transferability of our findings to rural or non-tertiary hospital settings. In terms of strengths, our study includes being one of the few qualitative studies on this topic in sub-Saharan Africa. We intentionally included the perspectives of women who had disengaged from HIV care. Our findings also contribute to theory by expanding our understanding of key differences between barriers to retention during pregnancy and after birth.

## Conclusion

Our findings suggest that interventions to improve postpartum women’s retention in HIV care should be multifaceted and may be most effective when simultaneously decreasing the economic burden associated with accessing ART, minimizing stigma, and improving clinical management of women who experience ART-related side-effects. Among other strategies to minimize economic barriers, hospitals could further decrease the frequency of refills for ART in the postpartum year from three to 6 months for women who do not need special monitoring, as some of our study participants recommended. Further, a family-based model of care, which not only provides maternal and child health, pediatric HIV, and postpartum HIV care services in one physical location, but also synchronizes these services and treats families as partners, may minimize the overall financial burden of accessing HIV care for mothers and thereby improve retention [35].

Our study suggests that the disproportionate burden of lower HIV retention rates in the postpartum year compared to in pregnancy is shaped by economic and social forces, rather than a lack of maternal motivation or less interaction with the healthcare system. Applying our knowledge of these factors (e.g. newborn care, HIV-stigma, and transportation costs) and their interactions can ultimately be used to inform the development of multi-faceted interventions to improve women’s retention in HIV care.

## Data Availability

The transcripts of the interviews are available upon reasonable request from the Center for Learning and Childhood Development, Ghana, P.O. Box 3190 AF, Accra, Ghana. Email: clcdghana@gmail.com

## Abbreviations

ART: Antiretroviral therapy
HIV: Human Immunodeficiency virus
MTCT: Mother-to-child transmission

## Footnotes

1 *Trotro* is an umbrella term for any privately owned, public, minivans that have frequent stops. Passengers can get in and out at any point along the van’s route. Because of the frequent stops, it often takes longer to get to one’s destination. Most vans do not have any air-condition system and they can be crowded and hot.
